# Developing a Healthcare and Medical School at King Faisal University: Implications for Educational Performance and Cost-Effectiveness

**DOI:** 10.3390/ejihpe13110168

**Published:** 2023-10-30

**Authors:** Ali Almomin, Abu Elnasr E. Sobaih

**Affiliations:** 1 Dental Clinics Complex, King Faisal University, Al-Hassa 31982, Saudi Arabia; aalmomain@kfu.edu.sa; 2 Management Department, College of Business Administration, King Faisal University, Al-Hassa 31982, Saudi Arabia; 3 Management Department, Faculty of Tourism and Hotel Management, Helwan University, Cairo 12612, Egypt

**Keywords:** healthcare, medical school, educational performance, cost effectiveness, King Faisal University, Kingdom of Saudi Arabia

## Abstract

This research explored the potential of developing a university healthcare and medical school within King Faisal University, Saudi Arabia, by merging four medical and/or healthcare colleges within the institution into a medical school. The objective of a medical school is to produce professionals who are capable of performing the most fundamental tasks in healthcare and medicine up to the expectations of the market. This research explored various case studies involving mergers, their importance, and how they were conducted to inform this research study. This study adopted a qualitative research approach to collect data from healthcare and medical colleges’ senior management, including deans, vice-deans, as well as heads of departments. The results showed several benefits of mergers, although some challenges were also raised. Culture, which some interviewees identified as change-resistant and risk-averse, was recognised as a key challenge for implementing mergers. Additionally, the need for generating funds for the merger projects was identified as a challenge at the start of the initiatives. Furthermore, compliance with regulations and laws at a public university is another challenge. This study identified the need for a strategic framework that builds on stakeholders’ involvement and proper communication and addresses the proposals’ repercussions. Some implications for educational performance and cost effectiveness were highlighted.

## 1. Introduction

King Faisal University (KFU) is one of the oldest and most prestigious higher education and research institutions in the Kingdom of Saudi Arabia (KSA), established in 1975 AD [1]. The university has four colleges related to healthcare and/or medicine. These colleges are the College of Dentistry, College of Clinical Pharmacy, College of Medicine, and College of Applied Medical Sciences. This research explores the potential of developing a single healthcare and medical school at KFU. The medical school would accommodate the four colleges in performing their essential duties, i.e., to instil the profession’s ideal, teach and transmit knowledge, information and skills to students, and engage in scientific research and community service activities [2]. Hence, the purpose of the medical school would be to prepare quality graduates in various medical colleges and provide them with the required skills to begin medical practice [2].

The healthcare and/or medical sector aims to improve the health, well-being, and quality of life of those it serves, and each medical specialist chooses a career path to serve humanity in a selfless and beneficial way [3]. Therefore, millions of people turn to teaching hospitals for specialist procedures, lifesaving care, and sophisticated therapies. Additionally, this includes critical community services such as checkups, consultations, and treatment, which are always available in teaching hospitals [2]. University healthcare departments combine medical teaching staff, students, researchers, and patient care in a one-of-a-kind setting where the future generation of doctors, nurses, and other health professionals is educated [2,3]. University healthcare departments have four general goals: proper training and mentorship, financial fairness, good health, and responsiveness to community expectations [3]. The successful achievement of these goals is determined by how well healthcare systems perform these four critical roles: service supply, resource generation, funding, and stewardship [3]. Research (e.g., [4,5,6,7]) has shown that planned mergers involve intentionally combining two or more organizations into a single entity to efficiently and effectively meet outside opportunities and challenges. Higher education institutions adopt a range of growth, repositioning, and expansion strategies, including horizontal and vertical integration, downsizing, mergers and acquisitions, market development, and evolution, to obtain an edge over their competitors [4]. Increased student enrolment, changing social interests and requirements, and meeting new problems and opportunities such as technologies, research, and training are all grounds for a merger in higher education [4,6]. Universities and other higher education institutions are under several internal and external pressures, which sometimes call for mergers to achieve competitiveness [6].

### 1.1. Examples of Some Merged Cases

This section includes a review of some case studies involving mergers, whether successful or unsuccessful (a full description of the cases is presented in the Supplementary Materials). The first case study is a mid-sized private institution which undertook a cost-cutting and financial audit exercise that resulted in various cost-cutting measures [8]. One of several techniques devised by the dean, who was tasked with cutting expenditures, was to combine three small departments into one broad, more comprehensive one. The department was organized in a way that brought everyone together [8]. For a more in-depth illustration, the original departments were small before the merger, with eight faculty members or fewer, yet they all had similar academic programs. One small undergraduate program was eliminated from one of the affected departments. The full-time professor responsible for this program departed before the disestablishment declaration, and a new faculty member was hired to complete the program for an additional year [8]. Three chairpersons were reduced to one and the number of administrative assistants was reduced, resulting in administrative savings. Following academic retirees who were not replaced, there was an increase in faculty savings. Overall, the merger increased financial oversight by creating a single department with suitably scaled operational and personnel budgets. As a result, the new department has many positive aspects, like more people being aware of all the programs the college offers. The new department’s relationships and cooperation with other academic departments within the institution have improved. The faculty is attempting to enhance educational delivery because of the merger by revising the curricula for each program and learning from a more varied group of colleagues [8].

The second case study involves merging three major medical universities in Iran, namely the Tehran University of Medical Sciences (TUMS), Iran University of Medical Sciences (IUMS), and Shahid Beheshti Medical University (SBMU) [9]. However, after 2.5 years, they split up. The university merger faced strategic, procedural, and structural challenges in terms of its context and human resources. Factors like the merger’s vision, for example, goals and motives, involvement of stakeholders, merger statement, service interruptions, information systems, and power balancing, played a role in the merger’s failure. The new structure, geographical distance, and organizational culture are factors that should have been considered during the merger. The reaction of the personnel, as well as their education, had a role in the merger’s failure. In conclusion, the merging of the three universities was unsuccessful and faced various challenges, such as the lack of involvement of stakeholders in the decision-making process, the timing of the merge and announcement, employee resistance, and unclear vision and goals. It was argued that if they involved the stakeholders, the merger could succeed since significant decisions (e.g., the integration of programs and curriculums) should be built with the involvement of key stakeholders [7]. Top executives from the three institutions should have developed a clear vision, mission, goals, objectives, values, and policies for the new institution and communicated them to the professors and employees. Lastly, employees play a significant role in each institution, and the institution’s success depends on them in one way or another. The universities would have involved the employees in the merging decision and made them feel comfortable to avoid the fear of losing jobs since mergers tend to make employees feel uncomfortable [7].

The third case study merging academic departments in a tertiary institution [10]. The study explored merging academic departments within a tertiary institution by looking at the case of two separately managed educational departments in Auckland, New Zealand. The merger was undertaken by a central institution that has mixed support from the departmental staff, with the goal of providing economic and financial benefits to the two departments. Whereas the two departments offer courses in different disciplines, the courses are for common academic programs. Department A has more qualified staff, a high number of course offerings, high research outputs, and a large number of students failing. On the other hand, Department B has more academic staff and a higher student-to-staff ratio than Department A. To implement the merger, the central institution appointed a new merger team from outside the two institutions to head the two departments. The merger team identified the existence of differences in terms of management styles, workload models for staff, work practices and cultural differences across the departments. Staff from Department B were moved to a building occupied by staff from Department A. The effect of cultural differences could be more pronounced in this merger because several factors can result in loss of separate identity, potential redundancies, and reduced future enrollment. The study utilized survey questionnaires to collect data on staff perceptions concerning the values existing in the two departments, along with values in the merged unit.

### 1.2. Benefits and Challenges of the Mergers in Higher Education

The analysis of the above cases and earlier research (see, for instance, [6,7,11,12,13,14,15,16]) on mergers and integration in higher education have shown that they have a wide range of positive consequences or benefits for the merged institutions. According to Skodvin [6], mergers and acquisitions broaden academic profiles, provide greater access to higher education services, diversify funding sources, improve curricula cohesiveness and standardization, and deepen the relationship between education and research. Mergers also improve academic collaboration, knowledge production, student enrollment, and graduation rates, minimize financial reliance on the government, deduct administrative costs, improve effectiveness and efficiency, and provide a service that meets national economic and social goals [6,11]. Mok [12] confirmed that competitiveness and efficiency in the global market are the key benefits of mergers. Ahmadvand et al. [11] argued that the main two benefits of mergers in higher education are efficiency and effectiveness. Other benefits include enhancing decentralization, implementing equity strategy, handling organisational fragmentation and encouraging bigger organisations. Thier et al. [13] identified uncertainty avoidance, staff stability and meeting cultural divides across organisations as core benefits of merging teaching hospitals. McGinnis et al. [14] confirmed cost effectiveness as a key benefit of merging higher education institutions. Gevitz [15] stated that mergers of academic institutions enhance the quality of their graduates. Bansal et al. [16] added that mergers enhance collaborative research, access to funding and quality of outcomes at the merged institutions.

On the other hand, research [11,13,14,17,18,19] confirmed that higher education institutions including teaching hospitals, confront challenges in their merging efforts in practice. These challenges include the high cost of merging, different cultures and employee alienation [6]. Ahmadvand et al. [11] identified three main challenges for unsuccessful mergers in universities: cultural inharmoniousness, differences in standards among institutions and geographical distance. Ahmadvand et al. [11] confirmed that these factors are crucial for successful mergers; otherwise, the mergers become unsuccessful, such as case 1 discussed above. Kang and Liu [19] found that scientific research performance was negatively affected by the mergers in higher education institutions in China. The same authors found that this was because of two main reasons or challenges: cultural integration and government interventions. These two factors were found to significantly affect the success of the merger. Thieret al. [13] argued that mergers should consider organizational and individual dynamics. McGinnis et al. [14] stressed the role of funding, culture and legislative action in supporting a successful merger, although these factors become challenges for the merger. Kastor [17,18] highlighted the role of leadership in making a successful structure and a new merger. Hence, earlier research on successful mergers [11,17,18,20] confirmed the significant effect of three main variables on successful mergers: committed leadership, robust communication, and effective process.

### 1.3. Purpose and Research Questions of the Study

As highlighted earlier, the purpose of the current study is to explore the possibility of merging four KFU colleges of medicine into a single healthcare and/or medical school. This research explores the implications of this integration and merger for both cost effectiveness and educational performance. This research also explores the challenges that might face such a merger and how these challenges could be addressed. To the best of the researchers’ knowledge, there is no published research conducted on similar cases in the context of developing countries such as the KSA, where a successful merger has been undertaken. Case 2 above was conducted in an Iranian context, but it was an unsuccessful merger due to some of the reasons discussed earlier. It is therefore important to undertake a new study in the context of the KSA to understand how a successful merger could be undertaken to contribute to the Saudi Vision 2030, which aims to make a radical change to the economy by encouraging other economic sources beyond the oil (further information could be accessed through: https://www.vision2030.gov.sa/en/ (accessed on 7 September 2023). This is especially important because it is expected that the medical school will bring various benefits to students, institutions and the community. First, it allows different colleges to bring their diverse cultures and identities together to develop unique organisations for students and the community. Second, the merger enhances the development, values, behaviour, and acquisition. Third, it promotes healthy competitiveness in the new organisation. Fourth, it improves the level of service quality provided to students. On the other side, many colleges and universities are under pressure to reach enrollment goals, despite rising expenses and overall financial issues while ensuring quality of education [8]. Students and their families expect first-rate facilities, cutting-edge technology, and excellent service. Educational experiences put financial pressure on institutions when they are already stretched to the limits. Therefore, it is crucial to understand the contribution of such mergers to both educational performance and cost effectiveness. The current research attempts to answer the subsequent research questions:

- What is the perception of the four medical colleges’ leadership regarding the integration and merger of their colleges into a single school?
- What are the possible challenges that confront such a merger and integration of these four medical colleges into one school?
- How this merger and integration could be implemented successfully while maintaining cost effectiveness and educational performance?

The following sections present the methods adopted for data collection and analysis and the results of the study. They are followed by a discussion and implications of the study. They are finalized the conclusions and the limitations of the study.

## 2. Methods

### 2.1. Research Approach and Research Paradigm

The study utilized a qualitative approach to investigate the potential of developing a university’s healthcare and medical school within KFU by merging four medical colleges. In order to achieve the purpose of this research and answer the questions, the qualitative approach was found more sufficient than the quantitative approach [21]. The qualitative approach was found highly effective in gathering rich data from participants and generating insights into the potential development of a university healthcare and medical school [21,22]. This research followed the guidelines given in the Standards for Reporting Qualitative Research (SRQR) [23]. The case study approach was found to be more appropriate for exploring the merger of the four colleges into a single school at KFU. The research followed Yin’s [24] single case study approach of KFU with embedded units of the four medical and/or healthcare colleges. This choice of a single case study approach enabled the researchers to question the benefits and challenges of the merger and explore the implications of this merger for cost effectiveness and educational performance [25]. A constructivist research paradigm was followed where knowledge was constructed based on the perspectives of participants and consolidated arguments from the literature review [26].

### 2.2. Data Collection Methods and Sampling

This article reports the results of the first phase of a study on the merger of the four medical colleges in a single school at KFU. It reports the results of in-depth, semi-structured, interviews with senior management of the four colleges (deans, vice-deans, and department chairs). The in-depth interviews enabled the researcher to collect sufficient information for understanding the case study and fulfilling the research objectives [27]. This article reports the results of 15 in-depth interviews with senior management of the four colleges. This number of interviews was confirmed after data saturation was reached [28], i.e., until interviewees reported the same information and there was consensus on the information provided by the interviewees.

The participants in this study were carefully selected based on their specialization and expertise in the intricacies of healthcare and medical education within KFU. The selection was accomplished through a rigorous process of purposive sampling. Special attention was paid to individuals whose voices and insights would add an invaluable dimension to the conversation. The inclusion criteria for these distinguished individuals centred on their willingness and eagerness to share their opinions and experiences relating to the multifaceted development of the healthcare and medical school within KFU.

The interviews were conducted face-to-face after the participants’ consent at their offices with the presence of the two researchers. Audio recordings were made and transcribed verbatim to ensure accuracy by the two researchers. Interviews were led by the supervisor of the research. This is because he has enough experience in conducting such interviews and participating in similar research projects. As discussed earlier, the present investigation leans heavily on the use of semi-structured interviews as the principal channel of data acquisition [27]. The interview encompasses a spectrum of intriguing open-ended questions and probing prompts to gain thought-provoking responses from participants. Notably, these questions were tailored and fine-tuned with the aid of a rigorous and systematic exploration of the research objectives. The interview guide underwent a meticulous pilot testing process with a judiciously selected group of participants to ensure lucidity and understanding of the questions. Issues discussed with interviewees came from the research questions and included discussing the merging proposal, their perceptions, challenges for this merger and recommendations for a better merger.

### 2.3. Data Processing and Analysis

Data analysis was conducted with meticulous attention to detail and rigor, using the powerful and dynamic tool of the qualitative research method. The research embraced thematic analysis to identify specific patterns and themes from the collected interview data. The researchers utilized a multifaceted approach, harnessing the full range of analytical techniques and strategies to ensure maximum insight and understanding of the data. The interviews were recorded and transcribed in full, after which the researchers engaged in a close and penetrating examination of the transcripts. Thematic analysis was employed to extract and identify the key themes and patterns, drawing on a wide array of theoretical frameworks and methodologies. Notably, this process involved multiple stages of iteration and refinement, with the researcher continually interrogating and re-evaluating the data to tease out its hidden nuances and subtleties. The analysis was conducted with the aid of advanced software tools, including the cutting-edge NVivo package, which provided unparalleled support and flexibility in the analysis process (the frequency of codes that emerged from the interviews is presented in the Supplementary Material file). The findings were then used to inform the study’s recommendations, which represented a groundbreaking and transformative contribution to the merger in the field of healthcare and medical education.

### 2.4. Ethical Guidelines

Ethical considerations were paramount in this study to ensure the utmost integrity and respect of the participants’ identities and rights. The researchers followed rigorous ethical standards and adhered to internationally recognized principles and procedures for human research. The study was approved by the ethical committee at the institution. Informed consent was collected from all participants, and the researcher provided comprehensive information on the study’s purpose, procedures, and potential risks and benefits. Measures to protect confidentiality and anonymity were strictly enforced, with participants’ identities safeguarded to prevent any breach of privacy. The study was culturally sensitive, considering the participants’ diverse backgrounds and perspectives. The researchers were vigilant in avoiding any potential harm or exploitation. Where participants expressed distress or discomfort, the researchers provided appropriate support and referrals to relevant services. Hence, ethical guidelines were incorporated into every facet of the study.

## 3. Results

### 3.1. Interviewee Perceptions about the Merger

There was a consensus among most interviewees that there is a need to merge the four independent medical colleges into a single school, which consequently led to a wide range of benefits or advantages. Interviewees report some benefits of mergers, such as that the merging process allows colleges to take the best elements of their previous organisational cultures, discard the aspects that do not work, and establish and build a new targets at the large entity. Furthermore, they argued that a merger enhances development and promotes best practices and acquisition. Additionally, a merger encourages healthy rivalry between the colleges that become one medical school with shared goals, which positively influences the overall performance. Some interviewees argued that having a medical school because of merging is expected to improve interdisciplinary teaching and research. As a result, it is expected to boost the quality of teaching and research operations at the merged colleges.

The deans argued that healthcare is constantly evolving and acknowledged the role of innovation in driving progress. They placed a strong emphasis on interdisciplinary collaboration and staying up-to-date with emerging technologies to enhance the quality of healthcare education and practice. They added that healthcare is a complex system with multiple stakeholders, including patients, providers, insurers, and government agencies. They acknowledged that effective communication and coordination between these groups are essential for delivering high-quality care and ensuring the success of the proposed merger. The majority of interviewees agreed that access to affordable and equitable care is a fundamental human right and argued that the proposed merger can be an opportunity to support marginalized populations and reduce health disparities. They suggested involving various stakeholders to ensure that the merger plan aligns with their needs. To conclude, there was consensus among most interviewees, particularly the deans, that a properly managed merging process can give the amalgamated institution a new board of directors and administration and the opportunity to strengthen leadership and build on their core competencies. Building on the core competencies at each college contributes to a successful merger.

### 3.2. Challenges of Merging the Four Medical Colleges

Deans and department chairs commented on the financial sustainability of the four colleges, particularly in light of the New University System at KSA, which encourages independence and self-financing. Thus, maximizing revenue and minimizing costs are necessary to provide high-quality care and invest in future growth. However, a merger requires funding at the beginning to undertake the project. Notwithstanding this, the interviewees confirmed that the availability of funds for a merger is considered a key challenge. Hence, they suggest exploring potential funding sources and strategies to support the merger.

Availability of funds was not the only challenge of the merger, as interviewees argued that healthcare is a highly regulated industry and emphasized the importance of compliance with laws and regulations. Additionally, they placed a strong emphasis on risk management and ensuring that the proposed merger complies with legal and ethical boundaries. As a result, interviewees suggested conducting a thorough assessment of the potential legal and regulatory implications of the merger. Moreover, deans, vice-deans and department chairs emphasized the importance of cultural competence and understanding the unique cultural and social contexts of the communities that healthcare organizations serve. They advocate for policies that promote cultural humility and inclusivity that address health disparities. One of the key issues relating to the culture that was noticed during the interviews was the resistance of some department chairs to the change and merger without clear justification for their resistance. However, this could be a result of change resistance due to the risk-averse culture of some Saudis.

Interviewees acknowledged that healthcare is deeply affected by global trends and challenges, such as population growth, climate change, and infectious diseases. As such, deans and vice-deans believed that healthcare organizations should prioritize sustainability and resilience, and adopt practices that mitigate the impact of these global challenges on healthcare delivery. Hence, they argued that before a merger, colleges should be prepared for this change to ensure a successful merger due to this risk-averse culture among some members of the colleges, which could be one of the key barriers to merging the four colleges. Hence, they commented on addressing such challenges by raising the awareness of stakeholders about the value of mergers and involving them in a such process to ensure its success.

### 3.3. Mergers and Planning Process Requirements

There was an agreement between interviewees that healthcare is a rapidly changing industry that requires continuous learning and adaptation. This emphasizes the importance of staff development and training to ensure that the organization can adapt to new challenges and opportunities resulting from the merger. Therefore, they suggested exploring training and development opportunities to support the new healthcare and medical school sector. Some of the interviewees added that healthcare is driven by technology and data. They believe that the effective use of technology can lead to more efficient and effective care delivery after the merger. Hence, they suggested exploring the opportunities to leverage technology and data to support the proposed merger and enhance healthcare education and practice.

Some department chairs argued that healthcare as a service industry focused on meeting the needs of patients and their families. Hence, a merger should place a strong emphasis on patient-centred care and ensuring that the patient experience is positive and supportive. There is a need to engage policymakers and government agencies in healthcare to ensure that the proposed merger aligns with their objectives and priorities. Additionally, collaboration between various stakeholders is an important requirement of a successful merger. Effective communication and collaboration between stakeholders are essential for providing patient-centred care. Thus, they suggested that it is important to involve providers, patients, and their families in the planning process to ensure that the proposed merger plan aligns with their needs and priorities.

## 4. Discussions

The results showed that the leadership of medical colleges and the vast majority of interviewees perceived the merger of the colleges into medical and healthcare schools as an urgent need to meet the opportunities, especially the New University System in the KSA. Aligning with several previous studies (e.g., [6,11,12,13,14]) the interviewees counted several benefits of this merger including creating a new organisational culture, promoting healthy rivalry, improving interdisciplinary teaching and research, enhancing the quality of outcomes and having shared goals. However, the results confirmed the challenges of undertaking this merger, which are in line with previous research [14,16,17]. The identified challenges include the availability of funds, compliance with laws and regulations, and resistance of individuals. The results are in line with the work of McGinnis et al. [14], who found the prime effects of culture, funding, and alignment with legislation on successful mergers. The culture of Saudis is mostly risk-averse, and hence, they may not engage in change management quickly without proper involvement [29]. This could be a challenge for leadership to involve and engage all individuals in the merging process.

The current study showed that the case of merging medical colleges at KFU could be similar to case 1 discussed earlier [8], which has a successful merger and achieved cost effectiveness as well as positive outcomes. However, case 2 provided good recommendations to avoid unsuccessful mergers such as leadership support and considering geographical distance [9]. Case 3 gave more instructions for successful mergers of two departments [10]. The current research identified the need for a comprehensive and well-planned approach to developing a medical and healthcare school at KFU. This requires conducting a thorough feasibility study prior to the implementation of the merger. This would involve assessing the resources, recognizing key challenges, and opportunities involved in establishing a medical and healthcare school, as well as identifying potential collaborators and sources of funding. In addition, it involves collaborating with other healthcare providers in the region to ensure a coordinated approach to healthcare delivery. Additionally, the importance of quality assurance, accreditation, and resource management emerged as important themes in the interviews. These suggest that careful attention should be paid to ensuring that the medical and healthcare schools are of high quality and are accredited and effectively managed.

### 4.1. Implications for Educational Performance and Cost-Effectiveness

The impact of merging four medical and healthcare colleges into a single school within KFU is expected to be significant in terms of both educational performance and cost effectiveness if the merger was undertaken successfully. By merging the medical colleges, the university can create a more unified and cohesive healthcare and medical school. This can lead to some positive impacts, including:

1. Improved resource allocation by combining resources. The university can reduce duplication of efforts and allocate resources more efficiently. This can lead to a more optimal use of resources, resulting in cost savings and improved outcomes. This aligns with the new strategic direction of the Ministry of Education in KSA, particularly the New University System, which promotes universities’ independence and self-financing.
2. Enhanced academic and research capabilities. Merging the medical colleges can lead to the creation of a more robust research and academic environment. This can lead to increased collaboration and sharing of expertise, resulting in a more innovative and impactful research program.
3. Improved student experience. This helps the university to create a more comprehensive and integrated curriculum. This could provide students with a more well-rounded and relevant education that prepares them for the evolving healthcare landscape.
4. Increased competitiveness by creating a more unified and cohesive healthcare and medical school. This helps the university to increase its competitiveness in the region and beyond. This can help attract top talent, students, and research funding.
5. Improved patient outcomes by creating a more robust research and academic environment. This helps the university to advance medical knowledge and technologies. This can lead to improved patient outcomes and a more effective healthcare system.

### 4.2. A Strategic Perspective

Any organization going through change or a merging process is likely to be uneasy, and there could be a lot of pressure if things are not identified clearly, duties are not assigned, or the plan to merge is not strategically set [20,30]. Medical colleges have to plan for this merging proposal to be successful. When the colleges come together, having a successful merger is significantly influenced by cultural and organizational factors, and willingness to cooperate. Hence, the leadership of the colleges should enhance their chances by incorporating change management and developing the merging proposal. Change management provides direction for the organization as it transitions from a current condition to an anticipated future state [30]. The essence of change management is the successful transfer of individuals and organizations to a new corporate structure [20]. The following steps could be followed for a successful merger:

Step 1: Set a vision and mission for the new school. The medical college’s development should be practically redesigned focusing on how the new school functions in terms of enhancing performance and financial sustainability.
Step 2: Set strategic goals, which should be carried out with organizational development principles in mind. The strategic goals should aim to maintain efficiency and effectiveness, improve productivity, boost employee morale, and enhance performance [5].
Step 3: Learn about cultures. It is important that a merger considers new organisational culture, starting with assessing cultural differences. After that, redesign the cultural systems to align them with the new medical school’s goals.
Step 4: Place these differences in the context of teamwork and integration. In this phase, dialogue is one of the most productive tactics. During this procedure, all issues should be outlined, clarification of all concerning parts, and modifications to the topics causing dispute should be made. By going through the process of building a communication channel, it will be easier to focus on engaging individuals while ensuring that issues are discussed and resolved [5]. This will not only reduce the likelihood of employee resistance to change, but it will also reduce any uncertainties or anxieties.
Step 5: Involve related business, students and employees. One of the key strategies is to create and keep track of an attitude assessment for all students and staff to gauge how they feel about the merger and acquisition and spot those struggling to fit in with the new culture. In order to make the transition to the new school culture as easy as possible, the medical school administration will be able to develop ways to lower stress and assist students and staff. A clear communication channel should be established to prevent miscommunication and misconceptions and give them answers to their inquiries. A formal framework, task force, or person with authority to address their issues should be established for this. Communication and expressing thoughts and concerns should be possible between all faculty members and students. To help allay any ambiguity, unease, or panic that a person may experience, all the colleges should provide sufficient justification and answers to all staff and students’ inquiries and explain the significance of the merger. After addressing employee needs and concerns and identifying those who are prepared for change, the leadership of the new school should undertake orientation and training (when needed) to ensure that the medical school’s staff members help it reach its strategic goals.

## 5. Conclusions

The results of this research showed that the idea of merging medical and healthcare colleges to develop a single medical and healthcare school has great potential, despite the identified challenges. These challenges include funding, compliance with laws and regulations, and change resistance of individuals. It is quite evident from the research findings that executing such a proposal requires a solid strategic direction supplemented with a proper strategic and operational plan. A proposal like this needs a feasibility study before its execution. Additionally, to ensure the success of this proposal, it requires the involvement of key stakeholders and teamwork. One of the key reasons for successful mergers is including key stakeholders when making decisions and communicating openly. This will help properly manage the change and deal with the risk associated with this change. If stakeholders are involved, the merger is more likely to succeed since effective decisions, such as integrating programs, should involve key stakeholders. Employees’ involvement in the early stage of the merger process makes them more likely to support the move. Proper communication with other stakeholders, such as students and related businesses, should also be involved since they play a significant role in the merger.

As highlighted earlier, this research reports the results of phase one of a large study on the merger of four medical colleges into a single school. The research undertook in-depth interviews with a sample of interviewees (15 participants) from the leadership of four colleges at KFU as a part of a qualitative research approach to explore the perceptions of the merger as well as the key challenges of this merger. Despite the research following the guidelines of SRQR, the research had a small sample in one public university (KFU) in KSA with no triangulation of the data. Hence, caution should be taken about the generalization of the findings to other contexts without further examination.

## Data Availability

Data are available upon request from researchers who meet the eligibility criteria. Please contact the corresponding author privately through e-mail.

## Supplementary Materials

The following supporting information can be downloaded at: https://rb.gy/r7xlw.
